# Skin-on-a-Chip for Cosmetic Safety Assessment: From Reconstructed Skin Models to Regulatory Translation

**DOI:** 10.1021/acsomega.6c08734

**Published:** 2026-10-23

**Authors:** Qian Zhu, Wenhui Liu, Xinjie Lv, Xixi Wu

**Affiliations:** † School of Architectural and Hydraulic Engineering, Jiujiang Polytechnic University of Science and Technology, Jiujiang 332020, China; ‡ 117774 School of Medicine & Nursing, Huzhou Normal University, No. 759, Erhuan East Road, Huzhou 313000, China; § Institute of Digital Intelligence for Public Health, Huzhou Normal University, No. 759, Erhuan East Road, Huzhou 313000, China

## Abstract

Cosmetic safety assessment increasingly relies on human-relevant nonanimal methods that support mechanistic interpretation and align with a defined decision context. Reconstructed human epidermis (RhE) supports accepted irritation and corrosion procedures. Reconstructed human skin (RhS) and full-thickness equivalents provide tissue-level comparators for dermal architecture, penetration, and recovery. These models are distinct comparator classes. Rather than cataloging skin-on-a-chip (SoC) architectures, this review assigns an appropriate comparator to each cosmetic-safety question. It then defines the residual uncertainty that may justify SoC testing. It also specifies required readouts and evidence thresholds for a prespecified decision. Vascular and immune modules, controlled topical dosing, and multiorgan coupling are considered only when they address unresolved time course, exposure route, tissue context, or mechanism. Decision-ready readouts require traceable calibration, explicit units, prespecified normalization, drift control, variability estimates, comparator agreement, and quantitative acceptance criteria. No reviewed skin SoC has broad regulatory acceptance for routine cosmetic-safety decisions. Translation therefore requires four distinct stages: analytical validation, biological validation, context-of-use qualification, and regulatory acceptance. Current evidence supports SoC mainly as a higher-tier complement when established methods leave a decision-relevant uncertainty.

## Graphical abstract

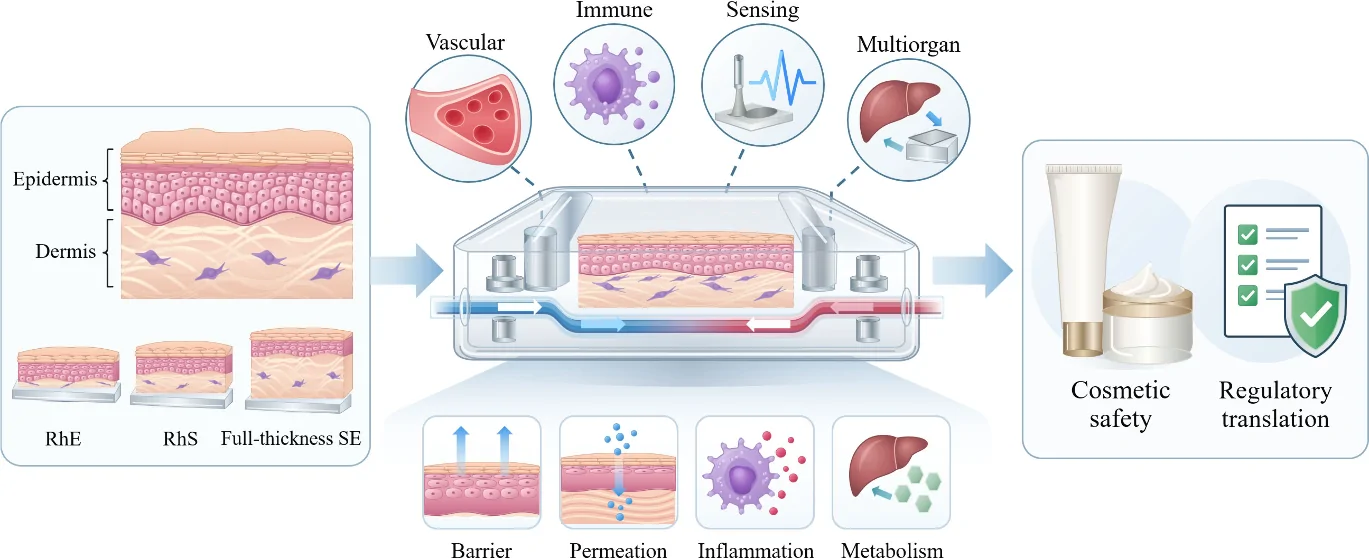

## 1 Introduction

Cosmetic safety assessment increasingly relies on nonanimal evidence. This evidence integrates physicochemical characterization, exposure assessment, in silico prediction, in chemico assays, and in vitro models. Within the European Union, Regulation (EC) No 1223/2009 restricts animal testing for finished cosmetic products and ingredients.^1^ Next-generation risk assessment (NGRA) frameworks organize human-relevant evidence for safety decisions.^2^ For skin-facing products, skin equivalents (SEs) provide human-cell-based test systems under controlled conditions. The principal comparator classes are RhE, RhS, and full-thickness skin equivalents. The Organisation for Economic Co-operation and Development (OECD) provides accepted Test Guidelines (TGs) for RhE-based skin irritation and corrosion.^3,4^ OECD TG 497 instead defines a mechanistically organized approach for skin sensitization.^5^ It does not prescribe one reconstructed-skin test. These uses establish the comparator framework for assessing whether SoC provides information beyond established methods.

Established SE models also define which questions require higher-tier testing. RhE supports standardized irritation, corrosion, and barrier end points. RhS and full-thickness equivalents add tissue architecture and dermal support. Static formats remain limited for resolving exposure history, vascular transport, immune-cell trafficking, neurocutaneous signaling, metabolism, and interorgan communication. These limitations matter for repeated application, formulation-dependent penetration, delayed barrier recovery, inflammatory amplification, vulnerable skin states, and route-dependent systemic or endocrine effects. Nanoenabled carriers, penetration enhancers, multiphase emulsions, antiaging actives, ultraviolet (UV) filters, hair dyes, and device-assisted delivery require time-resolved interpretation. A single viability or barrier end point may not resolve these effects. Reviews describe the relevant SoC architectures.^6,7^ A broader review situates them within the microphysiological system (MPS) landscape.^8^

Microfluidic SoC platforms can add controlled perfusion and compartmentalized topical dosing to SE models.^6,7^ Vascular and immune modules add transport and inflammatory functions.^9,10^ Neural cocultures, appendage-like organoids, and multiorgan coupling extend the range of mechanistic questions.^6,7^ These modules can measure barrier kinetics, vascular sink effects, immune activation, sensory mechanisms, tissue metabolism, and route-dependent exposure. A module is relevant only when it resolves a prespecified uncertainty that established comparators cannot address.

Previous reviews summarized SoC architectures and biomimetic artificial skin platforms.^6,7^ Related reviews addressed clinical translation and broader MPS standardization.^8,11,12^ This review instead defines the evidence required before a skin SoC result can inform cosmetic-safety decisions. It separates RhE, RhS, and full-thickness equivalents according to documented use. It assigns each SoC module to an unresolved uncertainty. It also distinguishes analytical validation, biological validation, context-of-use qualification, and regulatory acceptance. Evidence from cosmetic safety, skin-specific SoC, transferable sensors, and adjacent models is included only when its tier and limitations are explicit.

Figure 1 summarizes the sequence from regulatory drivers and comparator selection to SoC modules, standardized readouts, and qualification. The review begins with evidence identification (Section 2) and comparator selection (Section 3). It then addresses added biology (Section 4), readouts (Section 5), applications (Section 6), and regulatory translation (Section 7).

**Figure 1:**
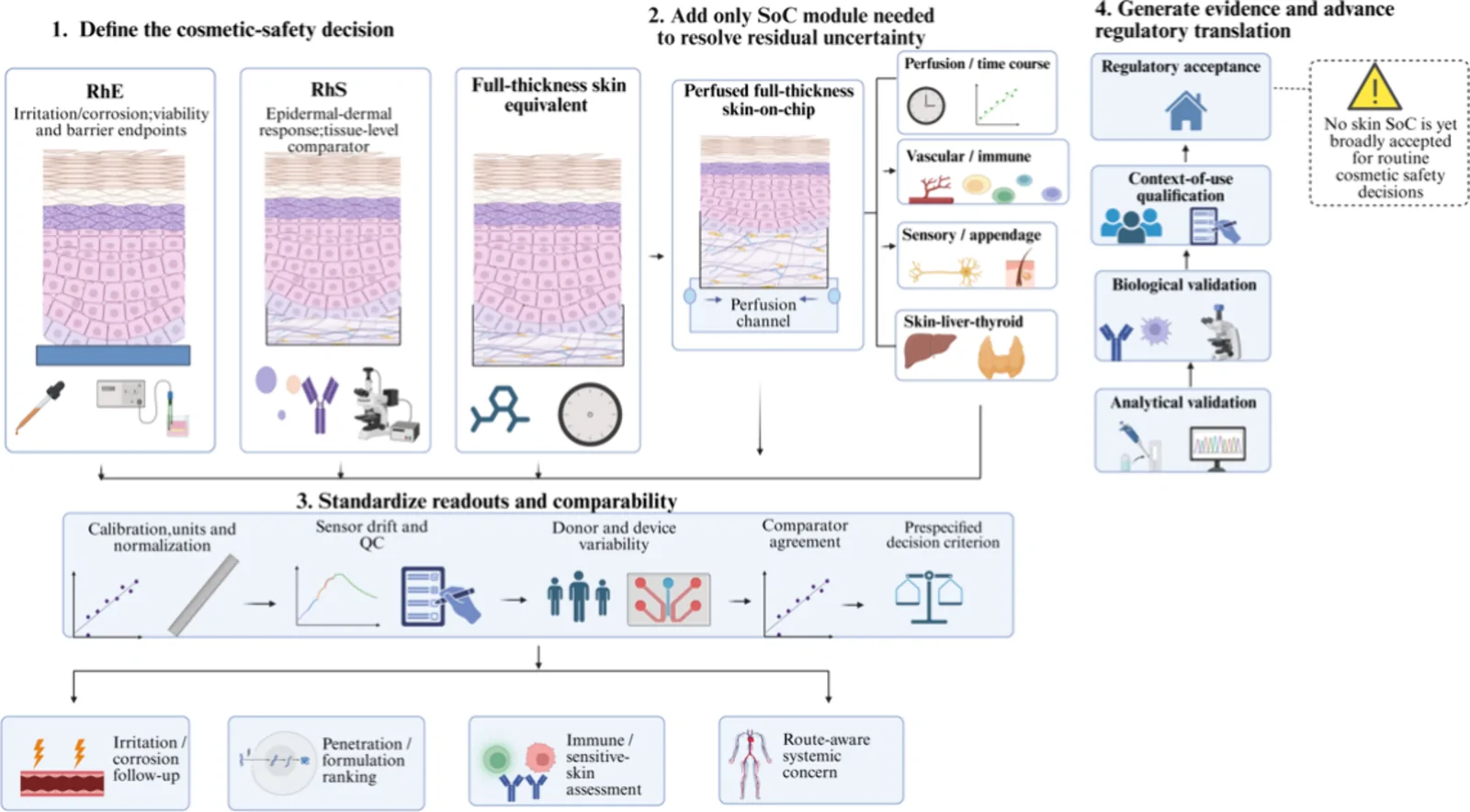
Decision-oriented framework for skin-on-a-chip (SoC) assessment of cosmetic safety. Reconstructed human epidermis (RhE), reconstructed human skin (RhS), and full-thickness skin equivalents provide distinct comparators. Additional SoC modules address residual uncertainty about exposure, transport, inflammation, sensory responses, or interorgan effects. Standardized readouts support analytical validation, biological validation, context-of-use qualification, and regulatory evaluation. Quality control (QC) is required throughout. The two component illustrations were created in BioRender and combined into this figure. Both BioRender publication licenses were obtained; license records are available at https://BioRender.com/2r94138 and https://BioRender.com/mg41zt7.

## 2 Evidence Identification and Study Selection

This structured narrative review used OpenAlex for primary retrieval. Backward reference checking and metadata verification in PubMed, Crossref, and publisher records supplemented the retrieval. The structured search was performed in May 2026 and updated on August 28, 2026. Eight query buckets combined core skin-on-a-chip or epidermis-on-a-chip terms with application or platform concepts. These concepts covered vascular or full-thickness models, cosmetic or dermal toxicity, disease contexts, bioprinting, organoids or appendages, on-chip sensing, and multiorgan coupling. The principal date window was 2020–2026. Studies from 2015 to 2019 were retained when they were methodological milestones. Older regulatory, material, or methodological references were retained only when they defined an accepted procedure or a persistent technical limitation.

The complete text strings recorded for the eight OpenAlex query buckets are reproduced below. Each query was submitted separately as a text string in OpenAlex; quotation marks below delimit the reported strings. The principal publication-year window was 2020–2026, with 2026 coverage ending at the August 28, 2026 update. Earlier methodological milestones and essential regulatory or materials references were identified through the supplementary reference checking described above.

- Q1 — Direct skin-on-a-chip systems: “skin-on-a-chip microfluidic skin”.
- Q2 — Vascularized or full-thickness models: “vascularized full-thickness skin-on-a-chip perfused skin model”.
- Q3 — Cosmetic or dermal toxicity: “skin-on-a-chip cosmetic toxicity irritation nanoparticle transdermal”.
- Q4 — Disease-context models: “atopic dermatitis psoriasis wound infection aging skin chip microfluidic”.
- Q5 — Bioprinting: “3D bioprinted skin vascularized microfluidic chip”.
- Q6 — Organoids or appendages: “skin organoid hair follicle melanocyte perfusion microfluidic”.
- Q7 — On-chip sensing: “skin organ-on-chip TEER impedance biosensor metabolite monitoring”.
- Q8 — Multiorgan coupling: “skin liver microphysiological system multi-organ chip topical pharmacokinetic”.

After title-level deduplication, 133 candidate records were screened. Sixty-two records were prioritized as the core experimental evidence set. Studies were included if they reported an experimental skin microphysiological platform or a reconstructed-skin comparator. Decision-relevant cosmetic or dermal applications were also eligible. Transferable measurement or qualification methods were included only when their evidence boundary was explicit. Reviews, editorials, conference abstracts, patents, and records with unverified metadata were excluded from the core set. Nonskin organ-chip studies were also excluded unless they had a transferable measurement or qualification role. Official guidelines and contextual reviews were retained when needed to interpret regulatory status and field-level standardization.

Detailed eligibility criteria were based on experimental content and evidentiary role. Eligible primary studies had to contribute to at least one of the following: (i) a skin or epidermal microphysiological platform with experimental evidence on tissue construction, perfusion, barrier function, vascular, immune, neural, or appendage-related function; (ii) a reconstructed-skin comparator relevant to irritation, corrosion, penetration, barrier monitoring, or tissue recovery; (iii) a cosmetic or dermal exposure study reporting toxicity, inflammatory responses, permeation, metabolism, or route-dependent effects; or (iv) a measurement or qualification method with a defined application to skin SoC. Disease, therapeutic, formulation, or device studies were eligible as adjacent evidence only when they informed a relevant biological module, exposure mechanism, or readout. Nonskin organ-chip and sensor studies required an explicit transferable measurement or qualification role.

Detailed exclusion criteria for the core evidence set were: duplicate records of the same study; reviews, editorials, conference abstracts, or patents; records whose bibliographic identity could not be verified; and nonskin organ-chip or general sensor studies without a defined transferable role. A skin-related clinical, therapeutic, or materials study without a clear contribution to the model, comparator, exposure, readout, or qualification framework was outside the experimental scope. Disease or therapeutic terminology alone was not a reason for exclusion. Official guidelines and contextual reviews were retained separately to interpret accepted procedures, regulatory status, and standardization; they were not treated as direct experimental cosmetic-safety evidence.

The 133-record candidate pool combined the structured retrieval with references recovered from the earlier manuscript and merged at title level. The 62 core records represented a priority set for detailed reading and evidence extraction, rather than the total number of references ultimately cited. Recent experimental studies in high-impact journals were prioritized, with directly relevant studies and methodological milestones retained outside this preference. Other eligible primary studies and essential background or regulatory sources could also support the narrative synthesis. Records outside the priority set were therefore not necessarily excluded from the review.

Each included source was assigned to one of five evidence tiers. The first three were direct cosmetic-safety evidence, skin-specific SoC evidence, and reconstructed-skin comparator evidence. The remaining tiers were transferable organ-chip or sensor evidence and adjacent therapeutic, formulation, device, or disease-model evidence. Only the first three tiers supported claims about current cosmetic-safety performance. Transferable and adjacent studies defined technical possibilities or evidence gaps. They are identified accordingly in the text. This classification prevents nondirect evidence from being interpreted as proof of cosmetic regulatory readiness.

Because the platforms, exposures, and end points were heterogeneous, no quantitative meta-analysis was attempted. The search and screening process was designed to make a critical narrative synthesis traceable, not to claim exhaustive coverage equivalent to a systematic review.

## 3 Application-Specific Comparator Framework for Cosmetic Safety

Comparator selection must follow the safety decision, not the generic label ″skin equivalent.″ RhE, RhS, and full-thickness skin equivalents differ in tissue complexity, documented use, and regulatory status. They cannot be treated as one comparator class. RhE is the accepted comparator for defined irritation and corrosion procedures. RhS and full-thickness equivalents are tissue-level comparators when dermal support, epidermal–dermal signaling, penetration, recovery, or long-term culture is required. Sensitization is evaluated through a defined approach rather than one reconstructed-skin model.

For irritation and corrosion, OECD TG 439 and TG 431 define RhE procedures, exposure conditions, controls, viability end points, and classification logic.^3,4^ A SoC result should therefore be compared with the corresponding RhE classification. Higher-tier SoC testing is justified only when the accepted assay leaves a prespecified uncertainty. Examples include borderline injury, repeated low-dose exposure, or recovery kinetics. Agonia et al. showed that RhE can support topical formulation comparison and barrier monitoring (Figure 2A).^13^ Their measurements included transepithelial electrical resistance and transepidermal water loss.

**Figure 2:**
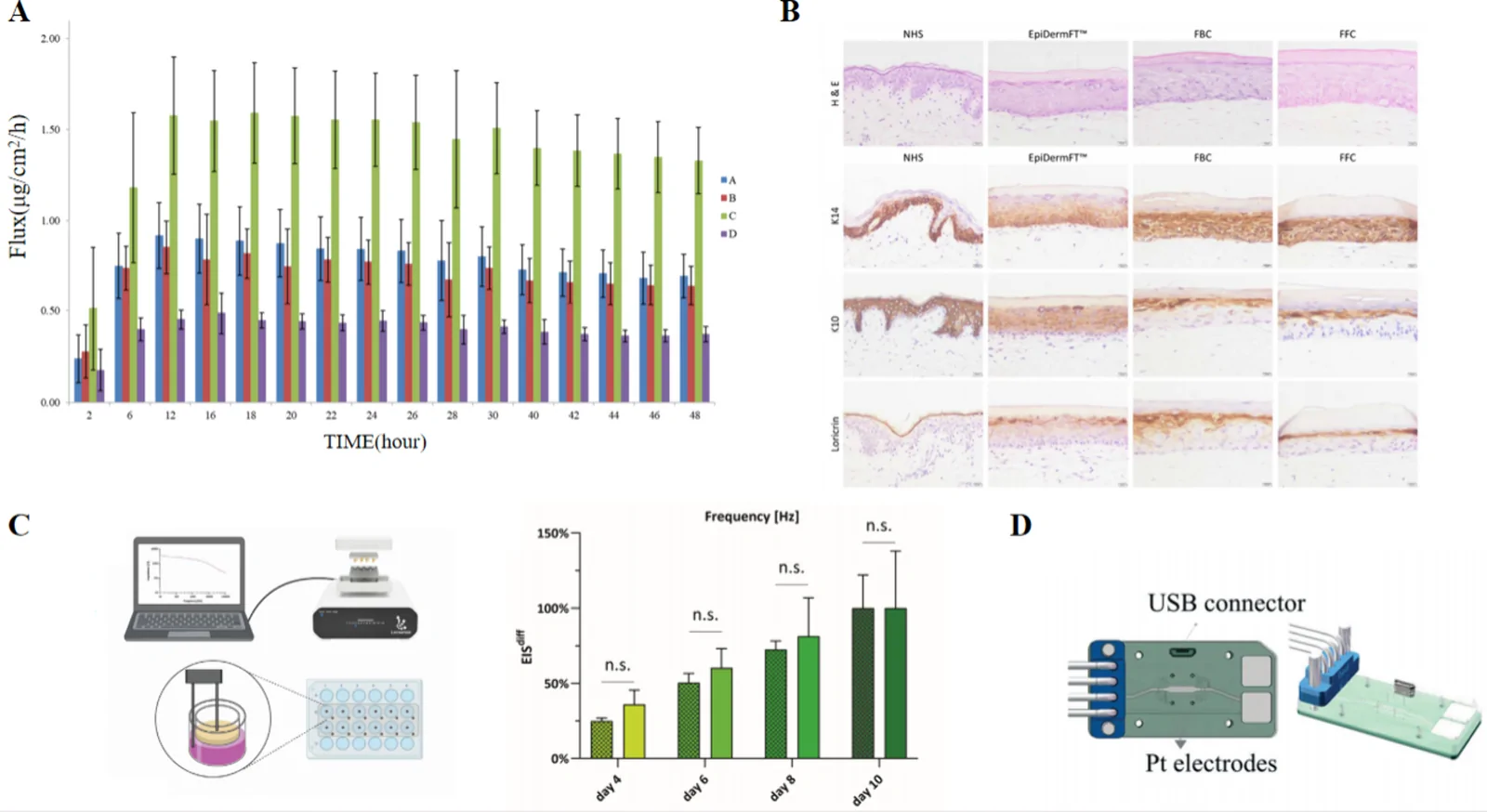
Reconstructed-skin comparators and dynamic testing. (A) Formulation-dependent flux measured using reconstructed human epidermis.^13^ (B) Histological comparison of a fish-collagen full-thickness skin equivalent with native and commercial skin models.^14^ Reprinted in part with permission from ref 14. Copyright 2024 Elsevier B.V. (C) Electrical measurement setup and barrier-development trajectory.^15^ Adapted with permission from ref 15. Copyright 2024 The Authors. (D) Microfluidic chip and electrode arrangement.^62^ Necessary permissions or applicable reuse licenses for published material have been obtained where required.

RhS and full-thickness skin equivalents add fibroblasts, extracellular matrix, and epidermal–dermal organization. This complexity does not confer the regulatory status of an OECD-accepted RhE method. Tan et al. compared a fish-collagen full-thickness equivalent with native human skin and a commercial model (Figure 2B).^14^ Van den Brink et al. used electrical impedance spectroscopy to quantify barrier development, defects, and repair in organotypic epidermis (Figure 2C).^15^ These models are appropriate when tissue architecture, dermal support, or longitudinal barrier function informs the decision. Tissue source, maturity, and end point definitions must be matched.

The comparison is therefore application specific. Irritation or corrosion follow-up is anchored to the relevant RhE procedure. Permeation or formulation ranking requires a documented transport method and tissue matched to the question. Immune or sensitive-skin assessment requires healthy and inflamed controls plus alignment with sensitization evidence. Systemic or endocrine interpretation requires exposure, toxicokinetic, and organ-function comparators. SoC is justified only when it resolves an uncertainty that the selected comparator cannot address.

## 4 Biological Dimensions Added by SoC Systems

Dynamic SoC architectures separate topical and basolateral or vascular-facing compartments. This design enables controlled medium movement, repeated dosing, basolateral sampling, and longitudinal exposure analysis. Salameh et al. constructed a perfusable full-thickness skin model with differentiated epidermal and vascularized dermal compartments (Figure 3A).^16^ They compared topical caffeine and minoxidil exposure with systemic benzo­[a]­pyrene exposure. Tárnoki-Zách et al. incorporated human skin equivalents into a controlled diffusion chamber for transdermal transport assessment.^17^ Two-organ studies extend this concept to toxicity beyond local irritation.^18^ Related skin-chip studies measured P-glycoprotein function and electrical barrier responses.^19,20^ Full-thickness and pumpless platforms support sustained culture or repeated exposure.^21–23^ Application studies used pumpless formats to evaluate α-lipoic acid and Curcuma longa extract.^24,25^ Adjacent formulation studies addressed adaptive nanocarriers, herbal extracts, and related transdermal systems.^26–28^ Nanobubble-assisted delivery provides a separate adjacent example.^29^

**Figure 3:**
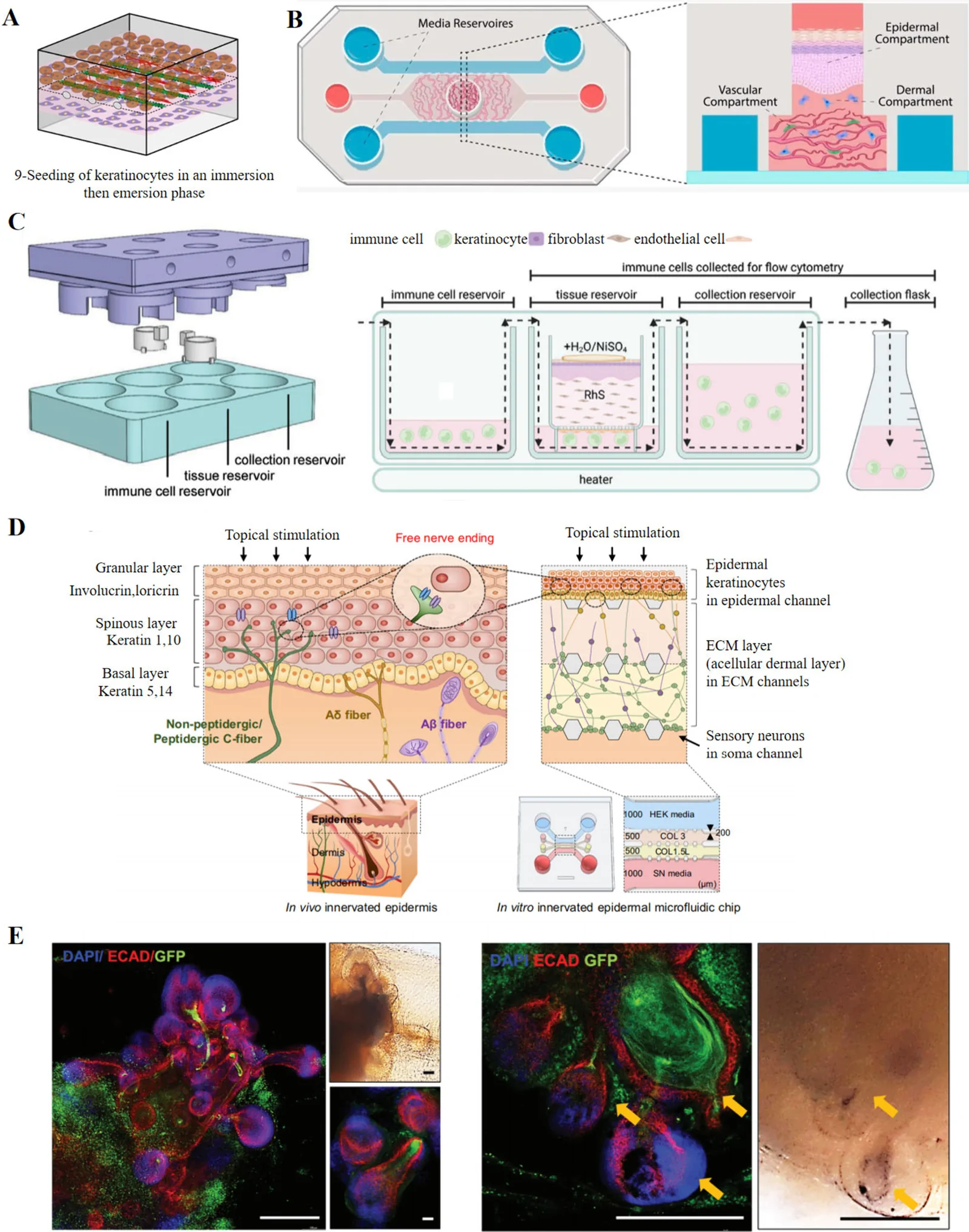
Biological functions added by SoC platforms. (A) Perfusable vascularized full-thickness skin.^16^ (B) Integrated microvascularized skin tissue.^30^ (C) Endothelialized skin chip supporting immune-cell flow and activation.^9^ (D) Innervated epidermal chip.^47^ (E) Microfluidic platform for skin-organoid differentiation.^48^ These examples illustrate distinct biological capabilities, not equivalent levels of cosmetic-safety validation. Adapted with permission from ref 48. Copyright 2024 Wiley-VCH GmbH. Necessary permissions or applicable reuse licenses for published material have been obtained where required.

Vascular modules add interfaces for nutrient exchange, vascular-side sampling, and circulation-facing exposure assessment. Jones et al. developed an integrated microvascularized skin equivalent containing epidermal, dermal, and vascular components (Figure 3B).^30^ Immune-competent platforms also resolve endothelial activation, immune-cell access, migration, and cytokine amplification. Ren et al. examined T-cell transmigration.^31^ Michielon et al. established controlled immune-cell flow in an endothelialized SoC system (Figure 3C).^9^ Kim et al. developed a contraction-free vascularized inflammatory architecture.^10^ Sun et al. modeled infection-associated immune recruitment in vascularized skin tissue.^32^ Integrated multiorgan-flow studies broaden the coupling design space.^33,34^ Bioprinted and stretchable vascular models add alternative tissue architectures.^35,36^ Angiogenesis, recirculation, and perfusable vascular channels support dynamic transport studies.^37–39^ Spatially patterned vasculature provides another engineering strategy.^40^ Inflammatory models define tissue-context and immune-response boundaries.^41–43^ Infection models provide adjacent evidence on immune recruitment.^44,45^ The clinical burden of skin disease motivates such models but does not establish cosmetic-safety performance.^46^

Neural, appendage-like, and organoid-enhanced systems address functions absent from standard reconstructed-skin assays. Ahn et al. developed an innervated epidermal-like coculture in a microfluidic device (Figure 3D).^47^ This format supports investigation of sensory-irritation mechanisms that viability or barrier damage cannot resolve alone. Quilez et al. used extracellular-matrix cues and morphogen gradients to generate skin organoids in a microfluidic device (Figure 3E).^48^ Related neural-delivery platforms add regenerative context.^49^ Hair-bearing organoids extend appendage modeling.^50,51^ Pigment-capable epidermal systems provide a separate comparator for pigmentation studies.^52,53^ These advanced modules remain more suitable for mechanistic investigation than routine cosmetic screening. Reproducibility, throughput, and comparator standardization remain under development.

Multiorgan SoC systems are relevant when an exposure question involves skin metabolism, liver metabolism, endocrine interaction, or route-dependent distribution. Dynamic skin-liver platforms examine pharmacokinetics, first-pass metabolism, and route-dependent responses to topical exposures.^54–56^ Skin-liver-thyroid systems add endocrine outputs under consumer-relevant conditions.^57^ Gut-skin and interconnected MPS studies provide transferable evidence for cross-tissue interaction.^58–60^ An integrated three-tissue platform illustrates an additional coupling strategy.^61^ These studies do not constitute direct cosmetic-safety evidence.

Each SoC module supplies a distinct capability. Perfusion enables dynamic exposure and sampling. Vascular compartments support transport and circulation-facing interpretation. Immune modules support analysis of inflammatory amplification. Neural and organoid systems support sensory and appendage-focused investigation. Multiorgan systems support toxicokinetic or endocrine interpretation. Figure 3 therefore aligns module selection with the unresolved cosmetic-safety question rather than biological complexity alone.

As summarized in Table 1, representative SoC architectures can be compared according to chip design, cellular composition, the biological model produced, and the principal measurements supported by each configuration.

**Table 1:**
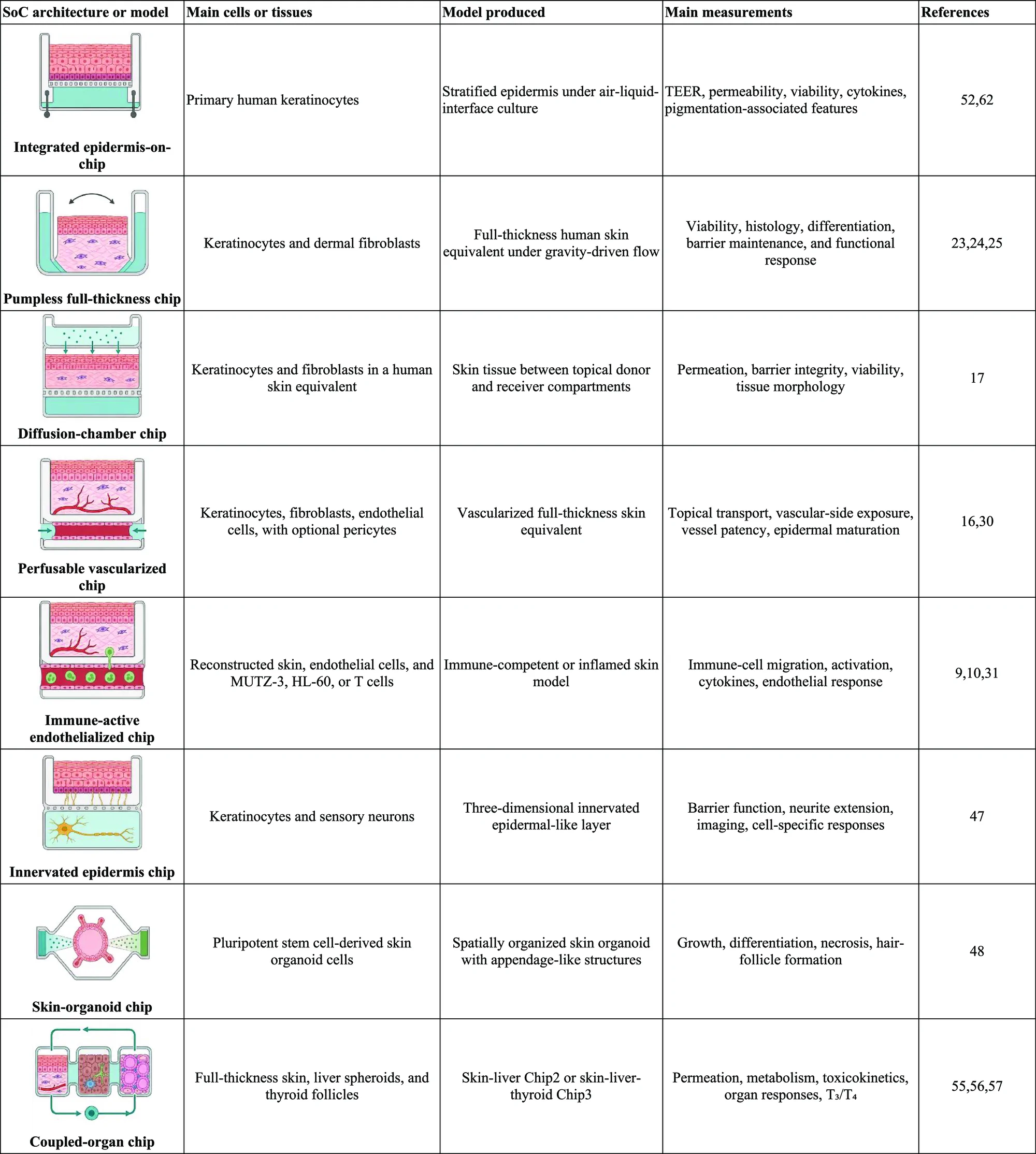
Representative Skin-on-a-Chip Architectures and Biological Models Relevant to Cosmetic Safety Assessment

## 5 Testing Parameters and Assessment Indicators

Testing parameters and readouts determine whether a SoC system generates interpretable cosmetic-safety evidence. Barrier integrity and permeability show whether a topical substance remains at the epithelial surface, disrupts the barrier, or reaches a lower compartment. The skin-specific Zhang platform combined transepithelial electrical resistance (TEER), permeability, viability, and inflammatory indicators.^62^ It is discussed as an irritation follow-up example in Section 6. Fernandes et al. demonstrated longitudinal impedance monitoring in a microfluidic epithelial model (Figure 4A).^63^ This nonskin study provides a transferable measurement strategy. Electrical and metabolic sensors support longitudinal measurements of impedance, oxygen, glucose, and lactate.^64,65^ Janiesch et al. distinguish general machine-learning methods from deep neural-network approaches.^66^ These concepts provide a methodological background. They do not establish monitoring performance in a skin chip.

**Figure 4:**
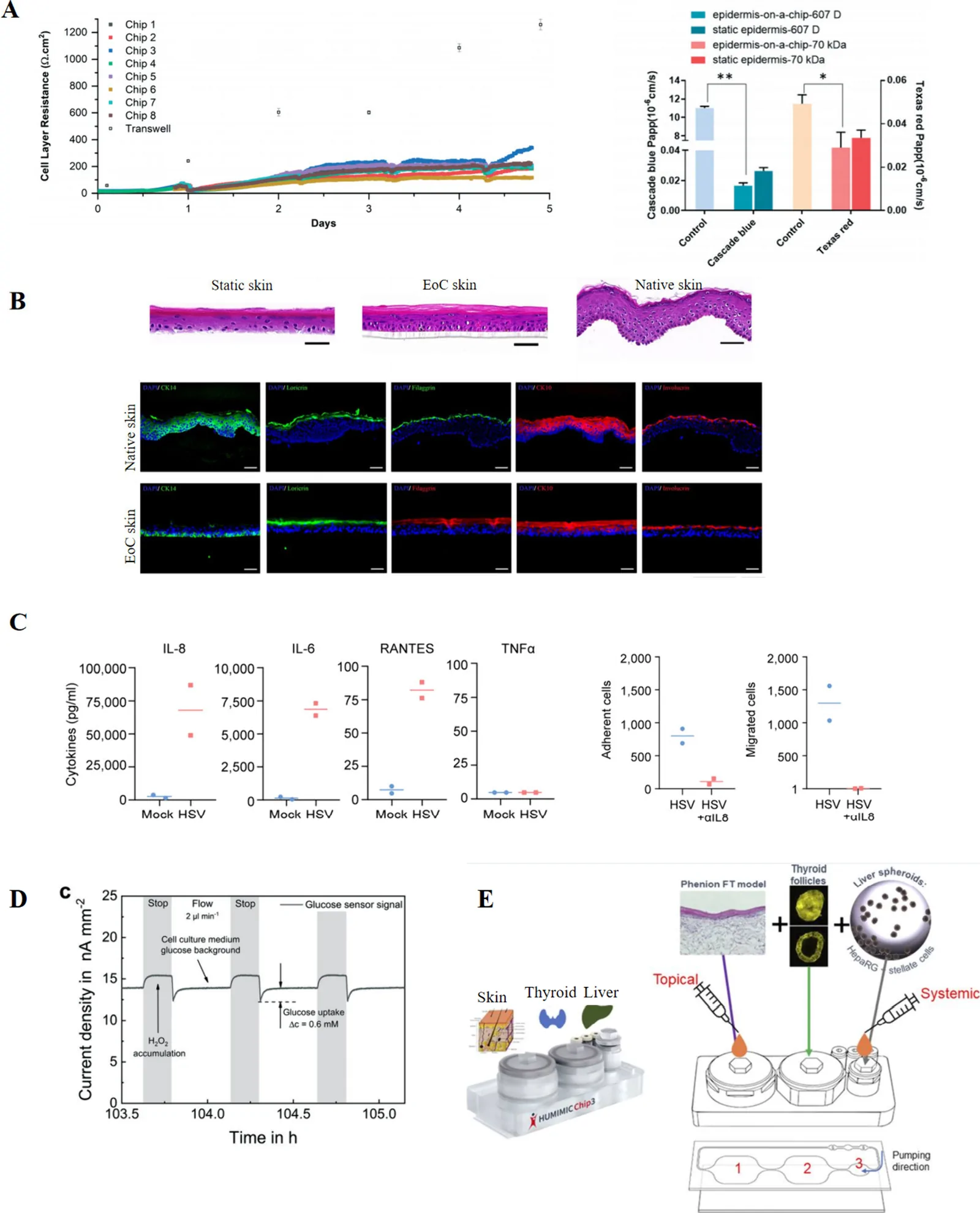
Representative structural and functional readouts. (A) Transferable epithelial impedance monitoring and skin-specific barrier measurements.^62,63^ Impedance component was adapted from ref 63. Copyright 2022 The Authors. CC BY 3.0. Skin-specific component was reprinted in part with permission from ref 62. Copyright 2021 The Royal Society of Chemistry. (B) Histology and epidermal differentiation markers in an epidermis-on-chip model.^52^ (C) Cytokine profiles and immune-cell recruitment after mock or herpes simplex virus exposure.^32^ (D) Glucose monitoring in a multianalyte microfluidic platform.^64^ (E) Skin–liver–thyroid coupling and hormone-analysis workflow.^57^ Necessary permissions or applicable reuse licenses for published material have been obtained where required.

Morphological and imaging-based indicators provide structural context for barrier and permeability data. Tissue thickness, epidermal stratification, differentiation markers, tight-junction organization, live–dead staining, immunostaining, and morphology distinguish barrier perturbation from cell loss or remodeling. The Li epidermis-on-chip and Ahn innervated coculture examples appear in Sections 4 and 6.^47,52^ These studies show why pigmentation and neuronal contact require complementary viability and barrier measurements. Figure 4B illustrates histology and epidermal differentiation markers in the Li model. These indicators are relevant when pigmentation, product color, sensory response, or remodeling could alter a viability-based interpretation.

Inflammatory and immune indicators are required when a substance may activate immunity rather than cause direct epithelial injury. Endothelialized and vascularized immune-active chips provide context for cytokine release, endothelial activation, and immune-cell migration.^9,10^ Sun et al. modeled herpes simplex virus (HSV) infection in a vascularized immune-competent skin chip (Figure 4C).^32^ The study quantified cytokine-associated recruitment and immune-cell behavior. This infection model provides mechanistic evidence, not routine cosmetic-classification evidence. Su et al. and Cognetti et al. developed integrated cytokine-sensing strategies that require skin-specific validation.^67,68^ Machine-learning-enabled multiplexed sensors provide another transferable architecture.^69^ Additional platforms integrate multianalyte microsensors or automated in situ monitoring.^70–72^

Metabolic, viability, and toxicokinetic indicators extend interpretation beyond visible tissue injury. Glucose consumption, lactate production, oxygen concentration, adenosine triphosphate content, mitochondrial stress, and long-term stability can reveal early tissue stress. Dornhof et al. integrated oxygen, lactate, and glucose sensors in a microfluidic platform. Figure 4D shows the glucose trace.^64^ This nonskin study provides a transferable continuous-monitoring strategy. Toxicokinetic interpretation requires measurements of parent-compound depletion, metabolite formation, skin or liver metabolism, and endocrine outputs. The skin-liver and skin-liver-thyroid systems in Section 6 define these readout classes (Figure 4E).^55,57^

The interpretive strength of SoC assessment depends on alignment between the readout and the safety question. Barrier and permeability measurements address irritation, penetration, and recovery. Imaging resolves architecture, pigmentation, and sensory morphology. Immune readouts address inflammatory or sensitization-related questions. Metabolic readouts address longitudinal stress. Toxicokinetic readouts address potential systemic or endocrine effects. Each readout requires calibration, reproducibility testing, documented operating conditions, and comparison with the selected tissue comparator.

A decision-ready readout pipeline has six sequential requirements. First, calibration must be traceable to the measurand and reported in explicit units. For electrical barrier measurements, report raw resistance, blank-device correction, electrode geometry, and area-normalized values such as Ω·cm^2^. Second, normalization must be prespecified relative to baseline, vehicle control, tissue area, cell number, or sampled volume. Third, establish sensor drift, carryover, and between-run quality-control limits with blank, negative, and positive controls. Fourth, separate donor, tissue-batch, device-lot, and operator variability. Report independent donor and batch counts together with variance components. Fifth, quantify agreement with the selected comparator using an end point-appropriate measure. Categorical end points may require concordance, sensitivity, and specificity. Continuous end points may require bias, limits of agreement, and rank consistency. Sixth, define an acceptance interval, an indeterminate zone, and a repeat rule before testing. Multiparametric data add value only when the pipeline converts instrument signals into a reproducible decision criterion.

## 6 Cosmetic-Safety Application Scenarios

SoC evidence becomes relevant when established comparators leave uncertainty about exposure kinetics, tissue recovery, inflammation, or route-dependent interpretation. For irritation, corrosion, and barrier perturbation, SoC systems are higher-tier follow-up tools. They are not replacements for OECD TG 439 or TG 431. Zhang et al. combined TEER, permeability, viability, and inflammatory indicators in an epidermis-on-a-chip model (Figure 5A).^62^ The platform distinguishes acute epithelial injury from barrier changes that develop or recover over time. Li et al. combined barrier and melanin-associated readouts in an epidermal microfluidic system.^52^ Pigmentation readouts are relevant when pigmentation, product color, photobiological stress, or photoactive ingredients may affect interpretation.^73^

**Figure 5:**
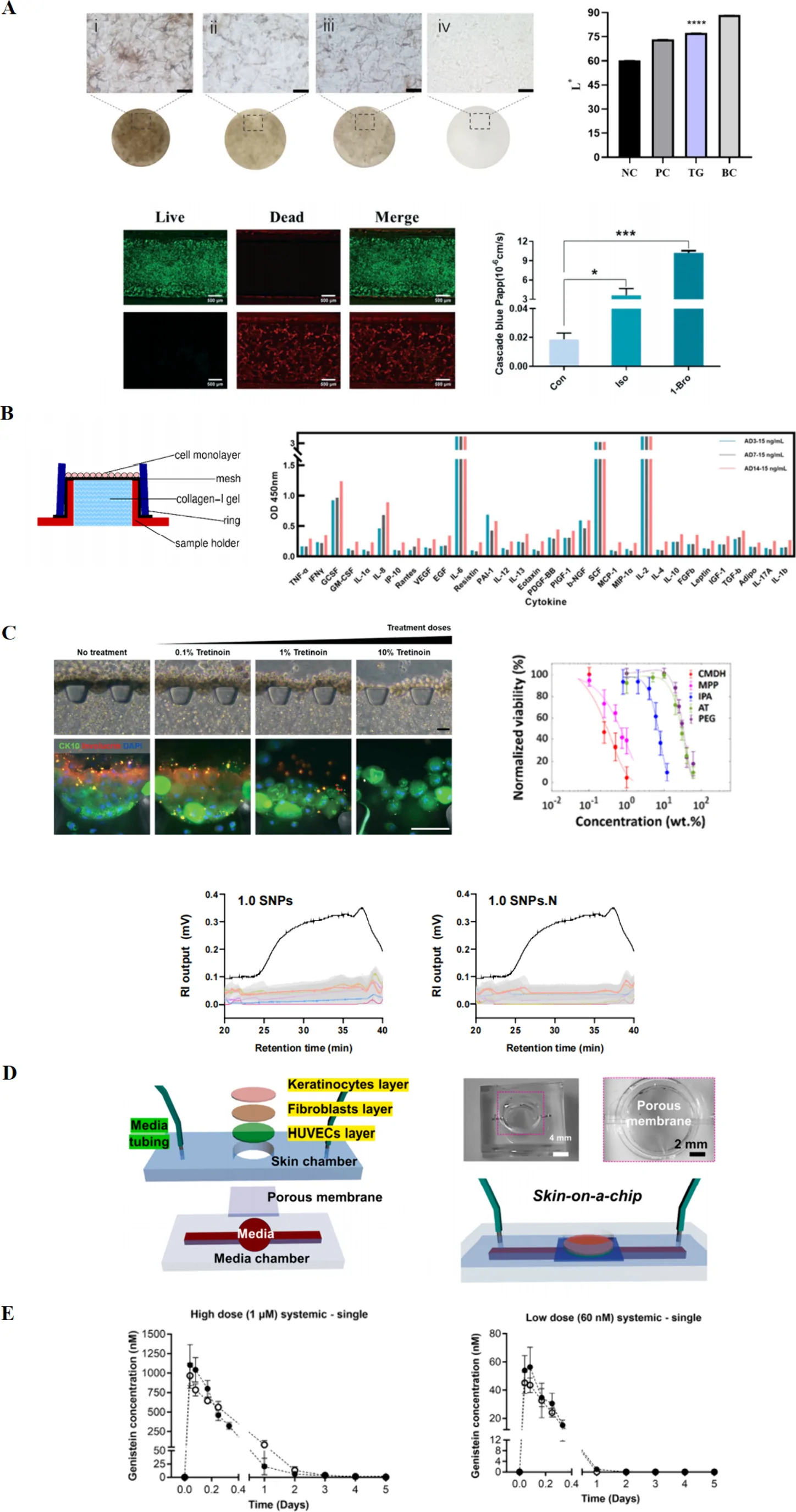
Application-relevant evidence from distinct skin models. (A) Melanin-associated readouts and selected barrier/viability measurements.^52,62^ Pigmentation component was adapted with permission from ref 52. Copyright 2023 The Authors. (B) Atopic-inflammation model and cytokine measurements.^45^ (C) Tretinoin-treated hybrid-skin images (upper left),^80^ spheroid-based concentration–viability curves (upper right),^77^ and starch-characterization chromatograms (lower panel).^76^ Reprinted in part with permission from refs 80 and 77. Copyright 2022, 2025 The Royal Society of Chemistry. These are separate studies, not a direct cross-platform comparison. (D) Microneedling-compatible skin-chip platform.^79^ Reprinted in part with permission from ref 79. Copyright 2024 The Authors. (E) Genistein concentration–time profiles after route-dependent exposure in a skin–liver Chip2 model.^56^ Reprinted in part with permission from ref 56. Copyright 2023 The Authors. Necessary permissions or applicable reuse licenses for published material have been obtained where required.

For sensitization, inflammation, sensitive-skin, or disease-context questions, SoC systems can provide complementary mechanistic evidence. OECD TG 497 organizes skin-sensitization evidence around an adverse outcome pathway (AOP).^5^ It does not rely on one reconstructed-skin test. Immune-flow and vascularized inflammatory platforms become relevant when immune access, endothelial interaction, or cytokine production affects interpretation.^9,10^ Kim et al. generated an atopic dermatitis-like skin equivalent using interleukin-4 and interleukin-13 (Figure 5B).^45^ Inflammatory and infection-response models define immune-activated contexts.^41,42,44^ Wrinkled and aged models define mechanically vulnerable states.^74,75^ Product-level conclusions require comparison with matched healthy controls.

Nanomaterials, penetration enhancers, complex formulations, and device-assisted delivery require formulation-specific exposure assessment. These systems can alter penetration, retention, local accumulation, barrier disturbance, and recovery. Kim et al. evaluated starch nanoparticles in human cells, artificial skin, and human skin.^76^ Figure 5C combines hybrid-skin treatment images, spheroid-based viability curves, and starch-characterization chromatograms. These panels represent distinct studies and should not be interpreted as a pooled comparison. Kalhori et al. used a spheroid-based SoC to rank small molecules and nanoparticles.^77^ Zhu et al. provided adjacent evidence on microfluidic formulation and penetration enhancement.^78^ Barros et al. developed a microneedling-compatible SoC (Figure 5D).^79^ The delivery studies are adjacent therapeutic evidence, but their exposure-control principles are transferable. Hybrid skin chips provide a closer example for chemical and cosmetic-compound testing.^80^ Microplastic, transferosome, and membrane-coated nanoparticle studies define particle-specific or formulation-specific concerns.^81–83^ Engineered skin and antiaging models extend tissue-level testing.^84,85^ Scaffold and high-throughput screening studies address model construction and formulation ranking.^86,87^ Cutaneous-toxicity models provide complementary experimental context.^88^ Chaturvedi et al. combined skin-chip permeation experiments with convection–diffusion modeling for caffeine and salicylic acid.^89^ This study supports compound-specific transport interpretation, not validated cosmetic classification. Microfluidic skin patches represent a device-associated exposure context.^90^

Systemic or endocrine concerns require interpretation beyond local skin responses. Hair dyes, UV filters, phytoestrogens, and other systemically active compounds may require assessment of metabolism, distribution, or hormone-related effects. Brandmair et al. compared reconstructed skin models in a skin-liver Chip2 system.^55^ The study showed that model selection can affect permeation and first-pass metabolic interpretation. A companion study examined route-dependent toxicokinetics and toxicodynamics of genistein (Figure 5E).^56^ Tao et al. extended the approach to a skin-liver-thyroid Chip3 model (Figure 4E).^57^ Gut-skin and interconnected MPS platforms provide transferable context for scaling and cross-organ interaction.^58–60^ Integrated three-tissue systems illustrate an additional coupling strategy.^61^ These applications remain exploratory because prespecified systemic criteria and regulatory comparators are absent.

Current evidence supports SoC as a higher-tier tool for defined cosmetic and skin-care scenarios. Relevant applications include barrier recovery, repeated exposure, inflammatory amplification, formulation-dependent penetration, vulnerable skin, device-associated perturbation, and route-dependent systemic interpretation. Routine irritation and corrosion classification remains anchored in established RhE procedures.^3,4^ Figure 5 shows where SoC may add evidence on time course, mechanism, exposure route, or tissue context.

## 7 Decision-Specific Regulatory Translation and Current Readiness

Translation begins with control of the delivered dose and exposure history. Polydimethylsiloxane (PDMS) can absorb small hydrophobic molecules and alter nominal-concentration interpretation (Figure 6A).^91,92^ This effect is relevant to fragrances, UV filters, dyes, lipophilic actives, and formulation excipients. Bubbles, evaporation, unstable flow, pump variability, device differences, and tissue contraction can also alter exposure. Pumpless designs reduce one source of operational variability.^23^ Contraction-free full-thickness designs improve tissue-shape stability during prolonged inflammatory culture.^10^ Stakeholder analyses identify control of these variables as a prerequisite for adoption.^93^ Broader organ-on-chip analyses likewise treat platform control as essential to translation.^94^ Qualification should therefore include material sorption, flow stability, bubble control, evaporation, and tissue geometry.

**Figure 6:**
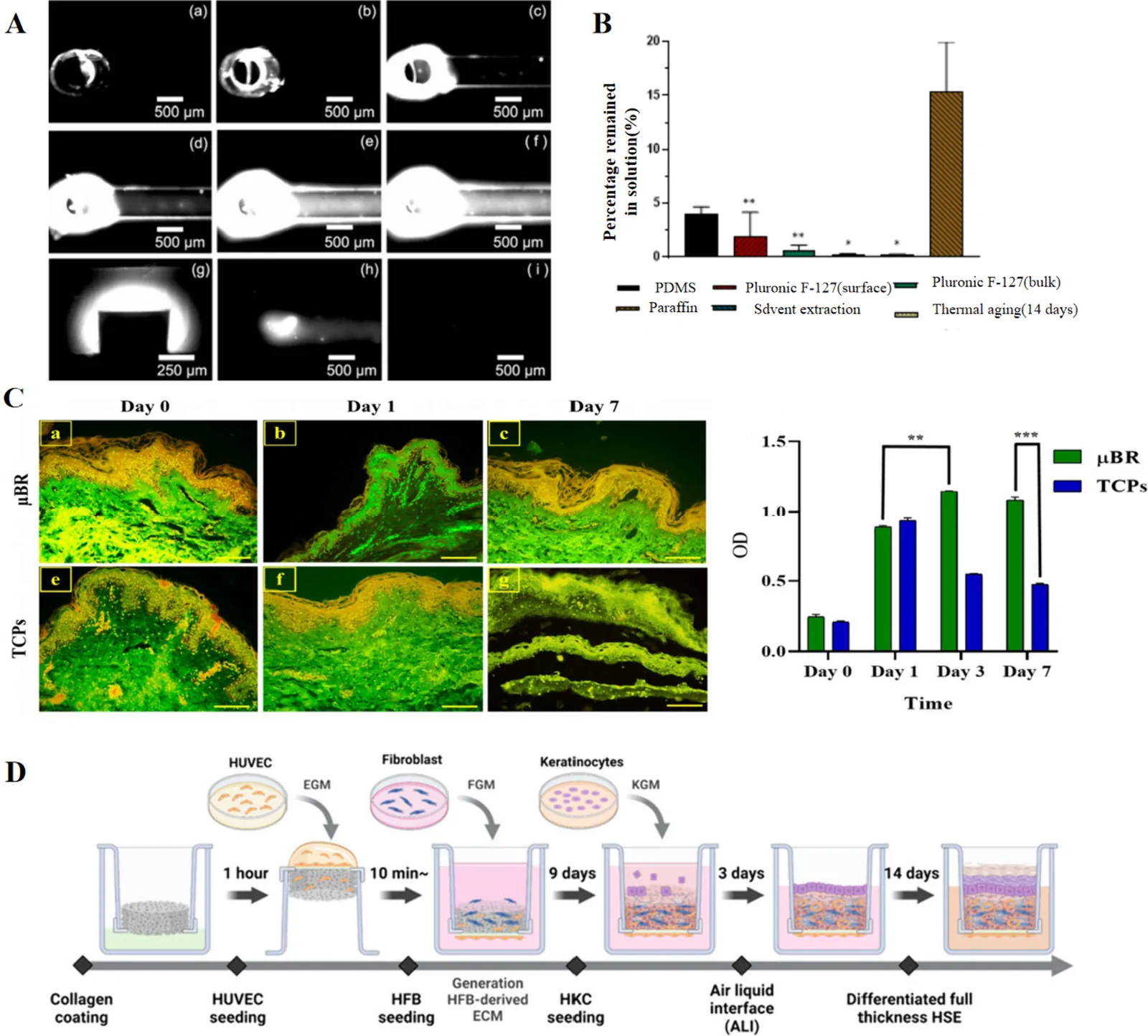
Material and platform challenges. (A) Small-molecule absorption by polydimethylsiloxane (PDMS).^91^ Reprinted in part with permission from ref 91. Copyright 2006 The Royal Society of Chemistry. (B) Partitioning of hydrophobic molecules in microfluidic materials.^92^ (C) Tissue viability in a pumpless skin-chip platform relative to tissue-culture plates.^23^ (D) Contraction-free vascularized full-thickness skin-chip platform. These examples illustrate exposure-control and reproducibility requirements.^10^ Necessary permissions or applicable reuse licenses for published material have been obtained where required.

Cell sources, tissue maturity, and comparator data sets determine whether SoC results can be interpreted across laboratories. Primary keratinocytes, fibroblasts, endothelial cells, immune cells, and induced pluripotent stem cell-derived tissues differ in biological state. Commercial RhE and RhS models also differ in donor background, passage number, barrier maturity, metabolism, and inflammatory responsiveness. These variables can alter responses to the same cosmetic ingredient. The skin-liver Chip2 comparison in Section 6 shows why overlapping uses require RhE or RhS comparator data sets.^55^ Skin-specific construction and culture studies show that fabrication and gel contraction affect viability and tissue geometry.^95,96^ Wound-healing models provide adjacent benchmarks for tissue maturity and repair.^97^ Reports should specify cell identity, donor number, passage number, tissue age, differentiation markers, and barrier maturity. Otherwise, apparent platform differences may reflect tissue composition rather than device performance (Figure 6D).

A conventional result should trigger SoC testing only when a prespecified residual uncertainty could change the safety conclusion. Reference sets must be application specific. Irritation or corrosion follow-up requires irritants, nonirritants, corrosives, and borderline formulations. Penetration ranking requires physicochemically diverse compounds and vehicles. Immune applications require inflammatory and noninflammatory controls. Systemic or endocrine questions require parent compounds and known metabolites. Dose, duration, repeated-application schedule, recovery interval, and sampling times must be defined before testing. Exclusion rules and decision criteria must also be prespecified. OECD TG 439 and TG 431 illustrate controlled procedures and performance criteria for irritation and corrosion.^3,4^ OECD TG 497 applies the same principle to defined sensitization approaches.^5^ SoC qualification should adopt this principle without assuming that one platform can serve every use.

Reporting and interlaboratory transfer determine whether SoC evidence can support use outside its originating laboratory. Reports should identify device material and lot, channel geometry, membrane or matrix composition, delivered flow, and shear. They should also identify cell source, donor, passage number, tissue age, exposure, and sampling protocols. Sensor calibration, normalization, drift correction, exclusion rules, excluded runs, and acceptance criteria must be reported. Independent laboratories should use shared reference materials and data templates. Current MPS adoption and standardization literature emphasizes terminology, measurement quality, reproducibility, and context-of-use performance.^6,8,93^ Low et al. discuss the validation and adoption challenges facing organ-on-chip technologies.^98^ Practical guidance also emphasizes fit-for-purpose design and transparent reporting.^99^

Practical adoption also depends on cost, access, throughput, and workflow compatibility. Cost per valid result includes fabrication, sensors, pumps or controllers, maintenance, and quality-control materials. It also includes matrices, cell sources, training, failed runs, and data analysis. Established RhE assays benefit from standardized kits and familiar workflows.^6,93^ SoC testing may require additional procurement, aseptic assembly, fluidic troubleshooting, and hands-on time. Pumpless and modular designs may reduce this burden.^23^ Simplified micromachined platforms illustrate one route to more accessible construction workflows.^100^ Scalability should be reported through batch size, hands-on time, failure rate, instrument occupancy, maintenance, and cost per accepted replicate. Institutional workflow inertia and validated legacy procedures may slow adoption. Economic feasibility must be evaluated for the intended decision and throughput. It should not be inferred from miniaturization alone.

Regulatory translation comprises four distinct stages. Analytical validation establishes calibration, selectivity, precision, drift control, reportable range, and data-processing rules.^101^ Biological validation establishes reproducible responses to reference chemicals and agreement with the application-specific comparator.^102^ Context-of-use qualification evaluates one defined safety question across independent runs, tissue batches, operators, and preferably laboratories.^102,103^ Regulatory acceptance is a separate authority-level determination. It cannot be inferred from publication, commercial availability, analytical validity, or a successful single-site study.^101,103^

No skin SoC identified in this evidence set has broad regulatory acceptance for routine cosmetic-safety decisions. Irritation and corrosion follow-up are closest to qualification because accepted RhE comparators and classification logic already exist. However, interlaboratory performance and criteria for added decision value remain unestablished. Permeation and formulation ranking may support internal decisions when dose recovery, mass balance, and comparator agreement are prespecified. Immune, vulnerable-skin, and multiorgan endocrine applications remain exploratory. Their reference sets, quantitative thresholds, and cross-laboratory predictive performance are not established. The pathway should start with an accepted or documented conventional result. A prespecified residual uncertainty must then justify SoC testing. The SoC must generate defined evidence, and a prespecified criterion must classify the outcome. The outcome may change the safety conclusion, support it, or leave it indeterminate.

Table 2 aligns each cosmetic-safety decision with its comparator, residual uncertainty, required readouts, prespecified criterion, and current readiness.

**Table 2:**
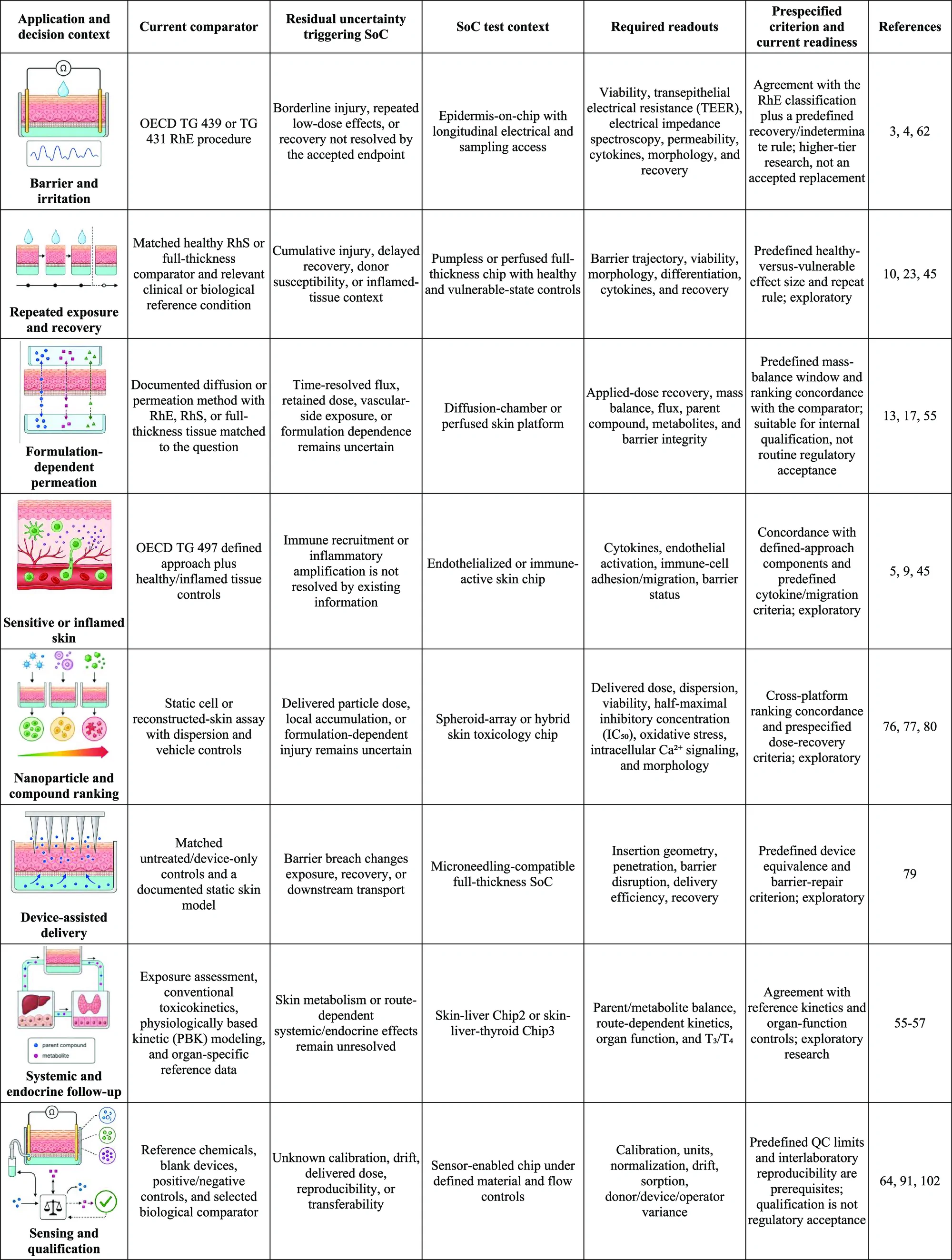
Representative Cosmetic-Safety Applications, Readouts, and Qualification Priorities for Skin-on-a-Chip Systems

## 8 Conclusions and Perspectives

Established methods provide the reference basis for cosmetic skin safety, and their roles are distinct. RhE anchors accepted irritation and corrosion procedures. RhS and full-thickness equivalents provide tissue-level comparisons for dermal architecture, penetration, and recovery. Sensitization is evaluated through a defined approach rather than one reconstructed-skin comparator. SoC is justified only when a documented approach leaves a prespecified uncertainty about time course, exposure route, tissue context, or mechanism.

The most mature current use cases are higher-tier investigations of barrier recovery, repeated exposure, formulation-dependent penetration, and inflammatory amplification. Other relevant contexts include vulnerable skin, device-associated delivery, and route-dependent systemic interpretation. Direct cosmetic-safety evidence remains smaller than the broader skin-chip literature. Transferable sensors, therapeutic platforms, and disease models define technical possibilities but do not establish cosmetic regulatory readiness.

Near-term progress depends on aligning each result with a defined decision. Adding biological modules without a context of use does not establish decision value. Calibration, normalization, drift control, donor and device variability, comparator agreement, and quantitative criteria should be specified before testing. Interlaboratory transfer and cost per valid result also require prespecified evaluation. The four-stage pathway comprises analytical validation, biological validation, context-of-use qualification, and regulatory acceptance. It provides a transparent basis for determining when SoC can supplement established cosmetic-safety methods. Current evidence does not support replacement of those methods.

## Supplementary Material



## Notes

Transparent peer review (PDF)
