# Supplementary material for: Skin-on-a-Chip for Cosmetic Safety Assessment: From Reconstructed Skin Models to Regulatory Translation
Source: ACS Omega. 2026 Oct 23;11(43):65985–6000. doi: 10.1021/acsomega.6c08734 (PMC13737838; doi:10.1021/acsomega.6c08734)
Supplement: Supplementary file 1 [file ao6c08734_si_001.pdf]

Response Letter

Manuscript ID: ao-2026-08734t

"Skin-on-a-Chip for Cosmetic Safety Assessment: From Reconstructed Skin Models to Regulatory Translation"

Dear Prof. Jiménez-Barbero:

We thank you and the three reviewers for the careful and constructive assessment of our Review. We revised the manuscript point by point. The revision focuses on literature-search transparency, comparator selection, readout standardization, regulatory readiness, practical adoption barriers, and repeated claims. We also clarify that skin-on-a-chip (SoC) systems are higher-tier complements. They are not accepted replacements for established cosmetic-safety methods.

Editorial and reviewer comments are shown in black, responses in blue, and quoted revised manuscript passages in red with yellow highlighting. Locations below refer to the submitted marked revised manuscript. Text revisions are identified by page and section, while figures and tables are identified by figure/table number and page. Page-and-section references are used because line numbering restarts on each page and may shift with final formatting.

Editorial Office and Submission Requirements

Editorial requirement 1: Submit a point-by-point response and a manuscript with all changes highlighted.

Response: We prepared this point-by-point response and a highlighted revised manuscript in which every added or substantively revised passage is shown in red font with yellow highlighting. Line numbering restarts on each page so that each location is traceable.

Editorial requirement 2: Submit a clean manuscript file in addition to the highlighted reviewer file.

Response: We have prepared a marked manuscript with revised text in red and yellow highlighting, and a matching clean manuscript. The marked copy is designated “Supporting Information For Review Only”; the clean copy is the revised main manuscript.

Editorial requirement 3: Confirm all funding sources and validated ORCID information in the submission system.

Response: We confirmed the funding information and reviewed the author information in the submission system. The revised manuscript identifies the Huzhou Normal University start-up grant RK30083. The available ORCID information for the authors has also been verified in the submission system.

The revised funding statement is:

> This work was supported by start-up grants from Huzhou Normal University (RK30083).

Location: Page 25, Funding section.

Reviewer 1

Comment 1: The review lacks a clear description of the literature search strategy and study-selection process, so it is unclear how studies were identified and how representative the literature is.

Response: We agree. The previous manuscript did not explain how the evidence base was assembled. We added a dedicated Evidence Identification and Study Selection section. It reports the retrieval source, update date, query framework, time window, screening counts, eligibility rules, evidence boundaries, and limitations of the narrative design.

The search and screening description was added as follows:

> This structured narrative review used OpenAlex for primary retrieval. Backward reference checking and metadata verification in PubMed, Crossref, and publisher records supplemented the retrieval. The structured search was performed in May 2026 and updated on August 28, 2026. Eight query buckets combined core skin-on-a-chip or epidermis-on-a-chip terms with application or platform concepts. These concepts covered vascular or full-thickness models, cosmetic or dermal toxicity, disease contexts, bioprinting, organoids or appendages, on-chip sensing, and multiorgan coupling. The principal date window was 2020-2026. Studies from 2015-2019 were retained when they were methodological milestones. Older regulatory, material, or methodological references were retained only when they defined an accepted procedure or a persistent technical limitation. After title-level deduplication, 133 candidate records were screened. Sixty-two records were prioritized as the core experimental evidence set. Studies were included if they reported an experimental skin microphysiological platform or a reconstructed-skin comparator. Decision-relevant cosmetic or dermal applications were also eligible. Transferable measurement or qualification methods were included only when their evidence boundary was explicit. Reviews, editorials, conference abstracts, patents, and records with unverified metadata were excluded from the core set. Non-skin organ-chip studies were also excluded unless they had a transferable measurement or qualification role. Official guidelines and contextual reviews were retained when needed to interpret regulatory status and field-level standardization.

Location: Pages 4–5, Section 2, “Evidence Identification and Study Selection.”

Comment 2: The manuscript mixes direct cosmetic-safety studies with disease, therapeutic, infection, drug-delivery, general organ-chip, and transferable sensor studies without explaining why they were included.

Response: We now classify every source by evidentiary role. Direct cosmetic-safety, skin-specific SoC, and reconstructed-skin comparator studies support performance claims. Transferable sensors and adjacent therapeutic, formulation, device, or disease models are used only to define technical possibilities or evidence gaps and are explicitly labeled as non-direct evidence.

The evidence-tier rule was added as follows:

> Each included source was assigned to one of five evidence tiers. The first three were direct cosmetic-safety evidence, skin-specific SoC evidence, and reconstructed-skin comparator evidence. The remaining tiers were transferable organ-chip or sensor evidence and adjacent therapeutic, formulation, device, or disease-model evidence. Only the first three tiers supported claims about current cosmetic-safety performance. Transferable and adjacent studies defined technical

possibilities or evidence gaps. They are identified accordingly in the text. This classification prevents non-direct evidence from being interpreted as proof of cosmetic regulatory readiness.

Location: Page 5, Section 2, "Evidence Identification and Study Selection."

Comment 3: Regulatory translation is insufficiently developed; the manuscript should state which platforms are closest to implementation, which remain exploratory, and which evidence gaps prevent cosmetic-safety use.

Response: We substantially rewrote the translation section. It now distinguishes analytical validation, biological validation, context-of-use qualification, and regulatory acceptance. It states that no reviewed skin SoC has broad regulatory acceptance for routine cosmetic-safety decisions. It also ranks current applications by readiness. Table 2 aligns each decision with its comparator, SoC trigger, required evidence, criterion, and readiness.

The regulatory-status statement now reads:

> No skin SoC identified in this evidence set has broad regulatory acceptance for routine cosmetic-safety decisions. Irritation and corrosion follow-up are closest to qualification because accepted RhE comparators and classification logic already exist. However, interlaboratory performance and criteria for added decision value remain unestablished. Permeation and formulation ranking may support internal decisions when dose recovery, mass balance, and comparator agreement are prespecified. Immune, vulnerable-skin, and multiorgan endocrine applications remain exploratory. Their reference sets, quantitative thresholds, and cross-laboratory predictive performance are not established. The pathway should start with an accepted or documented conventional result. A prespecified residual uncertainty must then justify SoC testing. The SoC must generate defined evidence, and a prespecified criterion must classify the outcome. The outcome may change the safety conclusion, support it, or leave it indeterminate.

Location: Page 22, Section 7, "Decision-Specific Regulatory Translation and Current Readiness."

Comment 4: The proposed advantages of SoC systems are repeated across sections, reducing reading fluency, and the reference base should be critically updated.

Response: We removed repeated generic statements and retained one section-specific synthesis for each major argument. The Conclusion now distinguishes direct evidence from transferable or adjacent evidence. It also states the conditions under which SoC may add decision value. We added the 2025 second edition of the Organisation for Economic Co-operation and Development (OECD) Guidance Document on Good In Vitro Method Practices and two 2024 microphysiological system (MPS) quality and standardization sources as Refs. 101-103.

The revised concluding synthesis states:

> Established methods provide the reference basis for cosmetic skin safety, and their roles are distinct. RhE anchors accepted irritation and corrosion procedures. RhS and full-thickness equivalents provide tissue-level comparisons for dermal architecture, penetration, and recovery. Sensitization is evaluated through a defined approach rather than one reconstructed-skin comparator. SoC is justified only when a documented approach leaves a prespecified uncertainty about time course, exposure route, tissue context, or mechanism. The most mature current use cases are higher-tier investigations of barrier recovery, repeated exposure, formulation-dependent penetration, and inflammatory amplification. Other relevant contexts include vulnerable skin, device-associated delivery, and route-dependent systemic interpretation. Direct cosmetic-safety evidence remains

smaller than the broader skin-chip literature. Transferable sensors, therapeutic platforms, and disease models define technical possibilities but do not establish cosmetic regulatory readiness. Near-term progress depends on aligning each result with a defined decision. Adding biological modules without a context of use does not establish decision value. Calibration, normalization, drift control, donor and device variability, comparator agreement, and quantitative criteria should be specified before testing. Interlaboratory transfer and cost per valid result also require prespecified evaluation. The four-stage pathway comprises analytical validation, biological validation, context-of-use qualification, and regulatory acceptance. It provides a transparent basis for determining when SoC can supplement established cosmetic-safety methods. Current evidence does not support replacement of those methods.

Location: Page 25, Section 8, "Conclusions and Perspectives."

Reviewer 2

Comment 1: The Abstract should clarify the contribution relative to existing SoC reviews and identify the main unresolved regulatory challenges.

Response: We rewrote the Abstract to foreground the application-specific comparator framework, evidence tiers, standardized readout pipeline, and four-stage translation pathway. The revised Abstract also states the current regulatory boundary explicitly.

The revised Abstract reads:

> Cosmetic safety assessment increasingly relies on human-relevant non-animal methods that support mechanistic interpretation and align with a defined decision context. Reconstructed human epidermis (RhE) supports accepted irritation and corrosion procedures. Reconstructed human skin (RhS) and full-thickness equivalents provide tissue-level comparators for dermal architecture, penetration, and recovery. These models are distinct comparator classes. Rather than cataloging skin-on-a-chip (SoC) architectures, this review assigns an appropriate comparator to each cosmetic-safety question. It then defines the residual uncertainty that may justify SoC testing. It also specifies required readouts and evidence thresholds for a prespecified decision. Vascular and immune modules, controlled topical dosing, and multiorgan coupling are considered only when they address unresolved time course, exposure route, tissue context, or mechanism. Decision-ready readouts require traceable calibration, explicit units, prespecified normalization, drift control, variability estimates, comparator agreement, and quantitative acceptance criteria. No reviewed skin SoC has broad regulatory acceptance for routine cosmetic-safety decisions. Translation therefore requires four distinct stages: analytical validation, biological validation, context-of-use qualification, and regulatory acceptance. Current evidence supports SoC mainly as a higher-tier complement when established methods leave a decision-relevant uncertainty.

Location: Page 1, Abstract.

Comment 2: RhE, RhS, and full-thickness skin equivalents cannot be treated as one comparator class; each SoC application should be compared with the appropriate method, uncertainty, readout, and decision.

Response: We agree and revised the comparator section and Table 2. Reconstructed human epidermis (RhE) is tied to accepted irritation and corrosion procedures. Reconstructed human skin (RhS) and full-thickness equivalents are described as tissue-level comparators without implying equal regulatory status. Sensitization is

anchored to the defined approach. Penetration and systemic questions use application-specific documented comparators.

The comparator distinction now reads:

> Comparator selection must follow the safety decision, not the generic label "skin equivalent." RhE, RhS, and full-thickness skin equivalents differ in tissue complexity, documented use, and regulatory status. They cannot be treated as one comparator class. RhE is the accepted comparator for defined irritation and corrosion procedures. RhS and full-thickness equivalents are tissue-level comparators when dermal support, epidermal-dermal signaling, penetration, recovery, or long-term culture is required. Sensitization is evaluated through a defined approach rather than one reconstructed-skin model. For irritation and corrosion, OECD TG 439 and TG 431 define RhE procedures, exposure conditions, controls, viability endpoints, and classification logic.[3,4] A SoC result should therefore be compared with the corresponding RhE classification. Higher-tier SoC testing is justified only when the accepted assay leaves a prespecified uncertainty. Examples include borderline injury, repeated low-dose exposure, or recovery kinetics. Agonia et al. showed that RhE can support topical formulation comparison and barrier monitoring (Figure 2A).[13] Their measurements included transepithelial electrical resistance and transepidermal water loss. RhS and full-thickness skin equivalents add fibroblasts, extracellular matrix, and epidermal-dermal organization. This complexity does not confer the regulatory status of an OECD-accepted RhE method. Tan et al. compared a fish-collagen full-thickness equivalent with native human skin and a commercial model (Figure 2B).[14] Van den Brink et al. used electrical impedance spectroscopy to quantify barrier development, defects, and repair in organotypic epidermis (Figure 2C).[15] These models are appropriate when tissue architecture, dermal support, or longitudinal barrier function informs the decision. Tissue source, maturity, and endpoint definitions must be matched. The comparison is therefore application specific. Irritation or corrosion follow-up is anchored to the relevant RhE procedure. Permeation or formulation ranking requires a documented transport method and tissue matched to the question. Immune or sensitive-skin assessment requires healthy and inflamed controls plus alignment with sensitization evidence. Systemic or endocrine interpretation requires exposure, toxicokinetic, and organ-function comparators. SoC is justified only when it resolves an uncertainty that the selected comparator cannot address.

Location: Pages 5–6, Section 3, “Application-Specific Comparator Framework for Cosmetic Safety”; Table 2, Pages 23–24.

Comment 3: Readout standardization requires detail on calibration, units and normalization, sensor drift, donor variability, comparator agreement, and quantitative acceptance criteria.

Response: We added a six-step decision-ready pipeline. It covers calibration, units, normalization, drift, donor and device variability, comparator agreement, acceptance criteria, an indeterminate zone, and a repeat rule.

The added readout-standardization paragraph states:

> A decision-ready readout pipeline has six sequential requirements. First, calibration must be traceable to the measurand and reported in explicit units. For electrical barrier measurements, report raw resistance, blank-device correction, electrode geometry, and area-normalized values such as $\Omega \cdot cm^2$. Second, normalization must be prespecified relative to baseline, vehicle control, tissue area, cell number, or sampled volume. Third, establish sensor drift, carryover, and between-run quality-

control limits with blank, negative, and positive controls. Fourth, separate donor, tissue-batch, device-lot, and operator variability. Report independent donor and batch counts together with variance components. Fifth, quantify agreement with the selected comparator using an endpoint-appropriate measure. Categorical endpoints may require concordance, sensitivity, and specificity. Continuous endpoints may require bias, limits of agreement, and rank consistency. Sixth, define an acceptance interval, an indeterminate zone, and a repeat rule before testing. Multiparametric data add value only when the pipeline converts instrument signals into a reproducible decision criterion.

Location: Page 14, Section 5, "Testing Parameters and Assessment Indicators."

Comment 4: The regulatory section should show how a conventional result triggers SoC testing, what evidence the SoC should provide, and what prespecified criteria would support a decision; analytical validation, biological validation, qualification, and acceptance must be distinguished.

Response: We adopted the requested sequence. A conventional result must first leave a prespecified residual uncertainty capable of changing the conclusion. The SoC then generates the minimum evidence defined in Table 2, and a prespecified criterion determines whether the result changes, supports, or leaves the safety conclusion indeterminate. The four evidence stages are now defined separately.

The four-stage distinction now reads:

Regulatory translation comprises four distinct stages. Analytical validation establishes calibration, selectivity, precision, drift control, reportable range, and data-processing rules.[101] Biological validation establishes reproducible responses to reference chemicals and agreement with the application-specific comparator.[102] Context-of-use qualification evaluates one defined safety question across independent runs, tissue batches, operators, and preferably laboratories.[102,103] Regulatory acceptance is a separate authority-level determination. It cannot be inferred from publication, commercial availability, analytical validity, or a successful single-site study.[101,103]

Location: Page 22, Section 7, "Decision-Specific Regulatory Translation and Current Readiness."

Comment 5: Correct the typographical and grammar errors: chin -> chip; Wu et al. provides -> Wu et al. provide; Czirok et al. provides -> Czirok et al. provide; and use sensitization consistently.

Response: The requested typographical and subject–verb corrections were made. We also checked author attribution against the cited articles. The relevant first authors are Tárnoki-Zách (Ref. 17) and Zhu (Ref. 78), rather than Czirók and Wu. American English "sensitization" is used consistently outside published titles. The table headers, cross-references, and scientific notation were corrected.

Representative corrected text includes:

Tárnoki-Zách et al. incorporated human skin equivalents into a controlled diffusion chamber for transdermal transport assessment.[17] Zhu et al. provided adjacent evidence on microfluidic formulation and penetration enhancement.[78]

Location: Page 7, Section 4 (Tárnoki-Zách et al.); Page 16, Section 6 (Zhu et al.).

Reviewer 3

Comment 1: Add an explicit heading to the first column of Tables 1 and 2 so that each row is clearly identified.

Response: We added the first-column headings "SoC architecture or model" to Table 1 and "Application and decision context" to Table 2. We corrected two misaligned Table 2 rows. We also standardized T_3/T_4, IC_{50}, and

Ca^{2+} notation and expanded physiologically based kinetic (PBK) modeling at first use. Every continuation page now contains the relevant table header.

Location: Table 1, Pages 11–12; Table 2, Pages 23–24. Table cells are identified by column heading and row.

Comment 2: Expand the limitations to include economic and practical barriers: platform and equipment cost, access to devices/reagents/cells/expertise, training, laboratory workflow inertia, throughput, and scalability.

Response: We added a dedicated paragraph that addresses the full cost per valid result, access and procurement, specialized training, failed runs and maintenance, compatibility with established RhE workflows, and the metrics needed to demonstrate throughput and scalability.

The practical and economic limitations were added as follows:

> Practical adoption also depends on cost, access, throughput, and workflow compatibility. Cost per valid result includes fabrication, sensors, pumps or controllers, maintenance, and quality-control materials. It also includes matrices, cell sources, training, failed runs, and data analysis. Established RhE assays benefit from standardized kits and familiar workflows.[6,93] SoC testing may require additional procurement, aseptic assembly, fluidic troubleshooting, and hands-on time. Pumpless and modular designs may reduce this burden.[23] Simplified micromachined platforms illustrate one route to more accessible construction workflows.[100] Scalability should be reported through batch size, hands-on time, failure rate, instrument occupancy, maintenance, and cost per accepted replicate. Institutional workflow inertia and validated legacy procedures may slow adoption. Economic feasibility must be evaluated for the intended decision and throughput. It should not be inferred from miniaturization alone.

Location: Pages 21–22, Section 7, "Decision-Specific Regulatory Translation and Current Readiness."

We again thank the Editor and reviewers for their careful comments. The revised manuscript clarifies evidence selection, comparator logic, standardized readouts, and regulatory readiness. The quotations and locations in this response refer to the submitted marked revised manuscript. The additional responses below clarify figure provenance, permission status, and submission requirements.

Additional Editorial Office Requirements — September 2026

1. Include email addresses for all corresponding authors.

Response: Xixi Wu is the sole corresponding author. Her email address, wuxixi1015@gmail.com, appears on the title page. Qi Zhu and Wenhui Liu are identified as co-first authors. Location: Page 1, title page.

2. Add complete credit lines and permissions for referenced material in Figures 2–6.

Response: We have obtained the necessary permissions or applicable reuse licenses for published material included in Figures 2–6. Source references and the relevant permission, copyright, or license information have been incorporated into the corresponding figure captions where required. Complete bibliographic details are provided in the reference list. Figure 2D has been traced to the original Zhang et al. (2021) study (ref 62), and Figure 5C identifies its three distinct source studies. Location: Figure 2, Page 7; Figure 3, Page 9; Figure 4, Page 15; Figure 5, Page 19; Figure 6, Page 21.

3. Clarify whether Figures 1, 2, 3, and 5 are author-created, public domain, or require permission.

Response: Figure 1 combines two component illustrations created in BioRender. Both BioRender publication licenses were obtained, and the corresponding license-record URLs are provided in the Figure 1 caption: https://BioRender.com/2r94138 and https://BioRender.com/mg41zt7. Figures 2, 3, and 5 contain material from the cited publications and are not presented as public-domain or wholly author-created images. Necessary permissions or applicable reuse licenses have been obtained where required, and the relevant source and permission/copyright/license information is provided in the corresponding captions. Location: Figure 1, Page 4; Figure 2, Page 7; Figure 3, Page 9; Figure 5, Page 19.

4. Clarify whether the photographs in Figure 4 were taken by the authors; otherwise provide credits and the ACS artwork/photograph rights agreement.

Response: The photographs and micrographs in Figure 4 were reproduced or adapted from the cited publications; they were not acquired by the authors of this review. Necessary permissions or applicable reuse licenses have been obtained where required, and the corresponding source and copyright/license information is provided in the Figure 4 caption. Location: Figure 4, Page 15.

5. Ensure that all table captions are visible; rotate Tables 1 and 2 if possible.

Response: Tables 1 and 2 are presented as editable tables on landscape pages. Their captions are visible above the tables, and column headings repeat on continuation pages. The scientific content and embedded illustrations have been retained. Location: Table 1, Pages 11–12; Table 2, Pages 23–24. Table cells are identified by column heading and row.

6. Include a Table of Contents graphic.

Response: The Table of Contents graphic was created by the authors in PowerPoint and is included on the final manuscript page under “For Table of Contents Only”. It is not a BioRender figure. Location: Page 36, “For Table of Contents Only”.

7. Upload the marked manuscript as "Supporting Information For Review Only".

Response: We have prepared both a marked manuscript and a clean version with identical text, figures, and tables. The marked manuscript uses red text with yellow highlighting and is designated "Supporting Information For Review Only". The clean manuscript contains no revision highlighting.

Figure 1 — revised artwork and caption

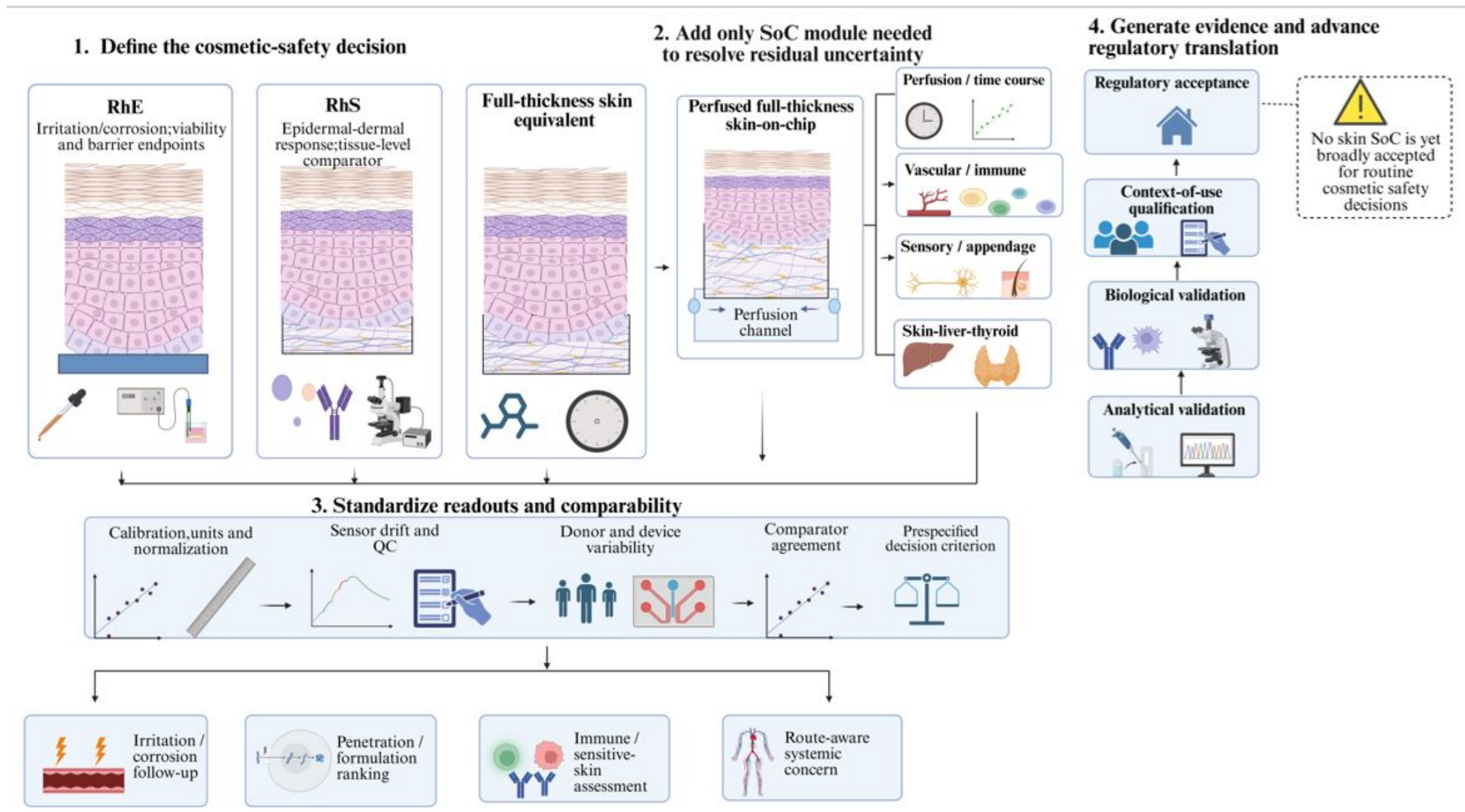

Figure 1. Decision-oriented framework for skin-on-a-chip (SoC) assessment of cosmetic safety. Reconstructed human epidermis (RhE), reconstructed human skin (RhS), and full-thickness skin equivalents provide distinct comparators. Additional SoC modules address residual uncertainty about exposure, transport, inflammation, sensory responses, or inter-organ effects. Standardized readouts support analytical validation, biological validation, context-of-use qualification, and regulatory evaluation. Quality control (QC) is required throughout. The two component illustrations were created in BioRender and combined into this figure. Both BioRender publication licenses were obtained; license records are available at https://BioRender.com/2r94138 and https://BioRender.com/mg41zt7.

Additional reference-integrity corrections

We corrected Ref. 66 to the authentic Janiesch et al. article, "Machine learning and deep learning" (2021), and limited its use to methodological background. The unverified Kim title at Ref. 89 was replaced by Chaturvedi et al., "In silico approach for validating organ-on-chips: exemplifying through a skin-on-chip device" (2026). Its cited claim now concerns compound-specific permeation modeling. The unverified Tagle title at Ref. 98 was replaced by Low et al., "Organs-on-chips: into the next decade" (2021), coauthored by Tagle. These are documented replacements, not claims that the original titles exist. We also removed a duplicate Ref. 1 entry. Location: Ref. 1, Page 26; Ref. 66, Page 31; Ref. 89, Page 33; Ref. 98, Page 34.
